# Effects of feminine hygiene products on the vaginal mucosal biome

**DOI:** 10.3402/mehd.v24i0.19703

**Published:** 2013-02-25

**Authors:** Bisiayo Fashemi, Mary L. Delaney, Andrew B. Onderdonk, Raina N. Fichorova

**Affiliations:** 1 Union College Class of 2016, Boston Latin Academy Class of 2012, Brigham and Women's Hospital Student Success Jobs Program Intern at the Laboratory of Genital Tract Biology, Department of Obstetrics, Gynecology and Reproductive Biology, Brigham and Women's Hospital and Harvard Medical School, Boston, MA, USA; 2 Laboratory of Genital Tract Biology, Department of Obstetrics, Gynecology and Reproductive Biology, Brigham and Women's Hospital, Boston, MA, USA; 3 The Anaerobe Laboratory, Department of Pathology, Brigham and Women's Hospital, Boston, MA, USA; 4 Harvard Medical School, Boston, MA, USA

**Keywords:** *Lactobacillus*, *L. crispatus*, cytokines, Interleukin-8, Nonoxynol-9, vaginal microbiota, vaginal epithelial colonization, mucosal immunity

## Abstract

**Background:**

Over-the-counter (OTC) feminine hygiene products come with little warning about possible side effects. This study evaluates *in-vitro* their effects on *Lactobacillus crispatus*, which is dominant in the normal vaginal microbiota and helps maintain a healthy mucosal barrier essential for normal reproductive function and prevention of sexually transmitted infections and gynecologic cancer.

**Methods:**

A feminine moisturizer (Vagisil), personal lubricant, and douche were purchased OTC. A topical spermicide (nonoxynol-9) known to alter the vaginal immune barrier was used as a control. *L. crispatus* was incubated with each product for 2 and 24h and then seeded on agar for colony forming units (CFU). Human vaginal epithelial cells were exposed to products in the presence or absence of *L. crispatus* for 24h, followed by epithelium-associated CFU enumeration. Interleukin-8 was immunoassayed and ANOVA was used for statistical evaluation.

**Results:**

Nonoxynol-9 and Vagisil suppressed *Lactobacillus* growth at 2h and killed all bacteria at 24h. The lubricant decreased bacterial growth insignificantly at 2h but killed all at 24h. The douche did not have a significant effect. At full strength, all products suppressed epithelial viability and all, except the douche, suppressed epithelial-associated CFU. When applied at non-toxic dose in the absence of bacteria, the douche and moisturizer induced an increase of IL-8, suggesting a potential to initiate inflammatory reaction. In the presence of *L. crispatus*, the proinflammatory effects of the douche and moisturizer were countered, and IL-8 production was inhibited in the presence of the other products.

**Conclusion:**

Some OTC vaginal products may be harmful to *L. crispatus* and alter the vaginal immune environment. Illustrated through these results, *L. crispatus* is essential in the preservation of the function of vaginal epithelial cells in the presence of some feminine hygiene products. More research should be invested toward these products before they are placed on the market.

## Introduction

Numerous women use feminine hygiene products every day; for some, it is part of their daily cleansing. Feminine hygiene products or methods may upset the normal pH level of ~4.5 in the vagina, which is important for maintaining the healthy vaginal immune barrier environment (1). Through the change of pH or through direct bactericidal properties, such products and practices may affect the composition of the normal vaginal microbiome, which is essential for the healthy mucosal environment and protection against yeast infection or other sexually transmitted pathogens. Vaginal lactobacilli are also important for having a healthy pregnancy and may prevent premature delivery and illness in the newborns (2). The disturbance of the normal microbiome, a condition called bacterial vaginosis (BV), is associated with significant risks for women's health as well as inflammatory complications in the newborn (3), while the presence of *Lactobacillus* has been associated with lower risk of inflammation (2). Lactobacilli and, especially, *L. crispatus* are among the bacteria that are most common in healthy women and characteristic for the healthy vaginal environment (4, 5). Lactobacilli are anaerobic bacteria that convert lactose and other sugars into lactic acid, which has a role in preventing infections (4). *Lactobacillus* is found in the gut and vagina, where it has anti-inflammatory and anti-cancer activities. In the industry, it is used to make yogurt and cheese, and it can also be used as a biotherapeutic. Because of its beneficial properties to the host, some *Lactobacillus* species have been used as probiotics available over the counter to aid mucosal health, for example, in the digestive tract. *Lactobacillus* products are also under investigation to restore vaginal health after prolonged use of antibiotics or to cure BV and prevent urinary tract infections (6, 7). *L. crispatus* has been found to effectively help inhibit the growth of harmful pathogenic microorganisms such as *Neisseria gonorrhoeae* (8). This study applied an experimental system for safety evaluation of vaginal products by testing their effects on *L. crispatus* growth and survival *in-vitro*. The evaluation was carried out by comparing products representing over-the-counter (OTC) douche kits, moisturizers, and lubricants to cell culture medium that maintains the growth of Lactobacilli *in-vitro* and nonoxynol-9 (N-9), a spermicide known to upset the vaginal immune barrier (9–11).

## Materials and methods

### Test products

The test articles listed in Table 1 (Vagisil Feminine Moisturizer, personal lubricant and moisturizer, and CareOne Douche) were purchased over the counter and were stored completely sealed, in their original box, at room temperature. The selection of products was made based on availability and public knowledge on each of the products. Once purchased, each product was screened to check for sterility. This was done by seeding each product undiluted over agar plates. None of the products showed any bacterial growth in this test. Phosphate-buffered saline (PBS) (Invitrogen by Life Technologies, Carlsbad, CA) and keratinocyte serum-free medium (KSFM) (Invitrogen, Carlsbad, CA) supplemented with epidermal growth factor, bovine pituitary extract, and CaCl_2_ as described (12) were obtained from Invitrogen, Carlsbad, CA. Nonoxynol-9 (N-9) was obtained from Personal Products Company (Skillman, NJ) (13) and diluted to 2% with KSFM. For assessing their effect on bacterial growth, all products were applied at 1:1 ratio over the bacterial suspensions.

**Table 1:** pH level along with ingredients listed on OTC product package

Products	Ingredients (from label)	Approximate pH
Vagisil Feminine Moisturizer	Water, glycerin, propylene glycol, hydroxyethylcellulose, DMDM hydantoin, diazolidinyl urea, disodium EDTA, polysorbate 20, methylparaben, propylparaben, *Anthemis noilis* (chamomille) flower extract, polyquatternium-5, citric acid, *Aloe barbadensi*, tocopheryl acetate	4
CareOne Douche	Purified water, benzoic acid, and vinegar	3
Personal lubricant	Propylene glycol, glycerin, *Trifolium pratense* flower extract (clover), methylparaben	5

### Preparation of bacterial suspension and treatment

*L. crispatus* was originally isolated from vaginal swab samples from healthy women participating in a vaginal microflora research study and then characterized and expanded in the laboratory of Dr. A. Onderdonk, Brigham and Women's Hospital (14). Once the volume needed was calculated, the frozen bacterial stocks were thawed in a room temperature water bath. The desired volume was transferred into a 50 mL tube and centrifuged down at 5000g× for 10 min at 25°C. After discarding the supernant, the bacterial pellet was resuspended in KSFM cell culture medium to the concentration of 7 × 10^6^ CFU/mL. Hundred microliter of the bacterial suspension was then added over 100 microliter undiluted product in 96-well tissue culture plates (Becton Dickson and Company, Franklin Lakes, NJ). The designated plates were then incubated on an orbital shaker in an anaerobic chamber (AnaeroPack System, PML Microbiologicals, Wilsonville, OR) at 35°C for 24 h or 2 h. From the 200 µl of test products along with bacterial suspension, three replicates within the same tissue culture plate were made. Each replicate contained 50 µl of the mixture of product and bacteria, along with 50µl of 50% glycerol, and was cryopreserved at −80°C.

### Dilution of test plates

To assess colony forming units, the bacterial suspensions in KSFM or in test product collected at each time point were serially diluted using 1× PBS. The first row of the 96-well plate contained *L. crispatus* along with the product. The following rows contained the desired amount of dilution in PBS achieved by transferring desired volume from one row to another using multichannel pipettes, and when the product had high viscosity, we used positive displacement pipettes (Gilson Medical Electronics through Fisher Scientific, Pittsburgh, PA).

### Seeding bacteria over agar plates

Brucella Agar Plates with 5% sheep blood, hemin, and vitamin K1 (BD, Franklin Lakes, NJ) were brought out from refrigerator to adjust to room temperature for 10 minutes. A pipette tip was used to mark either halves or thirds or the agar surface. After the serial dilutions of *L. crispatus* plus product were made, 30 µl was taken from a single well and was spread out over the agar, using a Steriloop (Fisher Scientific, Pittsburgh, PA). The plates were then incubated in an anaerobic chamber for 48–72 h, or until there were visible colonies.

### Colony forming units counts

Bacterial colonies were counted in the agar plates. The data was then entered into Excel, multiplied by the dilution factor, and transformed logarithmically (log10). The average log10 data from all dilutions that generated countable colony-forming units (CFU) for each culture in each experiment were used for statistical analysis of the triplicate cultures conducted in at least two independent seeding experiments for each time point and product.

### Preparation of vaginal epithelial cells and seeding over 96-well plates

We utilized for this study an immortalized vaginal keratinocyte cell line (Vk2/E6E7), which maintains the differentiation patterns and phenotypic characteristics of its normal human tissue of origin (12, 15, 16). Vaginal cells were grown in a T75 flask using KSFM medium. Once grown to confluence, the cells were trypsinized. After decanting the old medium, 5 mL of warm trypsin was added and the flask was tipped back and forth to wash away any dead cells. After removing the trypsin, 10 mL of fresh trypsin was added and incubated for 10 min at 37°C. After seven minutes, detachment of cells was checked under a microscope. Once enough cells were detached, equal volume of neutralization medium was added on top of the trypsin. The cell suspensions were then spun down at 500 g for 10 min. Next, the supernatant was decanted, and the cells were resuspended in 2 mL of antibiotic-free medium (AB-) and counted. The cell density was adjusted to 3 × 10^5^/mL and the suspension transferred to a sterile reservoir. Hundred microliter of suspension was transferred per a well into a 96-well plate. The plate was then incubated for at least 24 h before adding test products or bacterial coculture.

### Epithelial cell viability and IL-8 measurement

Following 24h incubation with undiluted and diluted test products, epithelial cell viability was assessed by MTT assay as described (17). IL-8 levels were measured in cell culture supernatants using a Meso Scale Discovery (MSD) small-spot immunoassay and Sector Imager 2400 (Meso Scale Discovery, Gaithersburg, MD).

### Bacterial-epithelial co-culture and epithelial-associated CFU counts

Details on the bacterial colonization model used in this study are published elsewhere (17). In brief, once the vaginal keratinocytes reached 100% confluence, medium was removed and replaced by 50 µL of bacterial suspension and 50 µL of test product. The plate was then placed in an anaerobic jar with one AnaeroPack inside and incubated for 24 h. After 24 h incubation, supernants were collected and frozen for cytokine assays. Using 200 µL of (1×) PBS, cell layers were washed in original plate twice with a multichannel pipette. Cells were then lysed by adding 100 µl of Hypure water and incubated at room temperature for 15 minutes followed by adding 100µl of (2×) PBS over the Hypure water and mixing well. Lysates were serially diluted in PBS and seeded over agar plates with a sterile loop. Agar plates were incubated under anaerobic conditions for 48–72 h, followed by CFU counting.

### Statistical analysis

Two-tail ANOVA analysis of variance and Prism (GraphPad Software Inc, San Diego, CA) were used to compare the CFU values, viability, and IL-8 levels obtained for test product and the control medium.

## Results

### Effect of products on planktonic L. crispatus culture

**Fig. 1:**
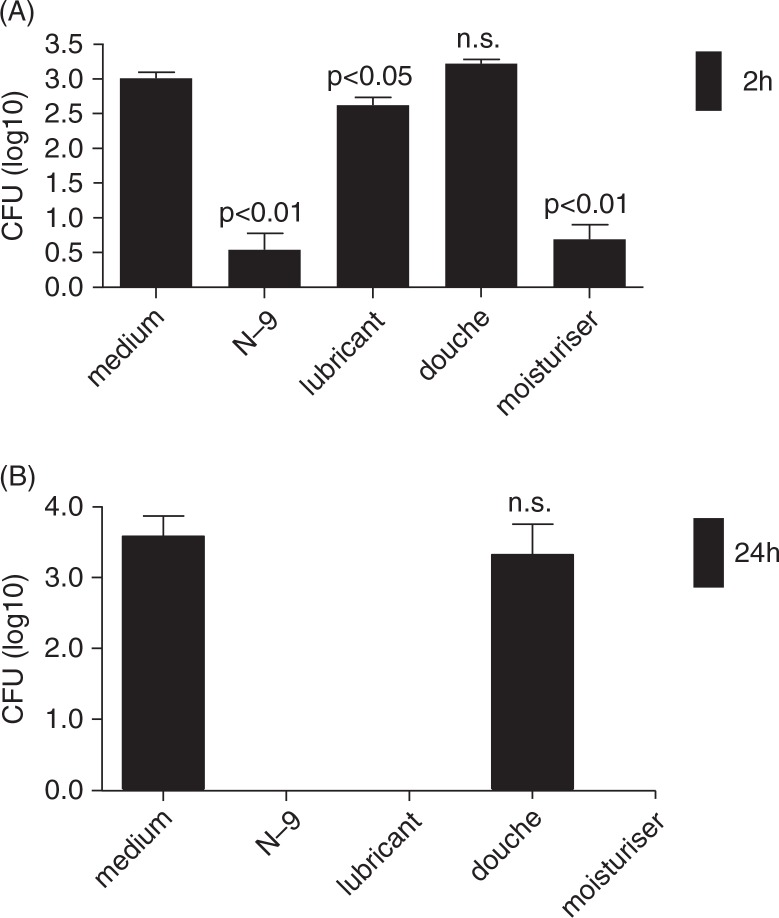
Composite effect of vaginal products on *L. crispatus* CFU. Bars represent logarithmically transformed means and standard errors of the mean from triplicate CFU measurements at (A) 2 h and (B) 24 h for each vaginal product exposed to bacteria twice. *P* values <0.05 and <0.01 show significant differences from the medium control. n.s. = no significant difference from control.

To simulate a naturally thriving environment of the natural microbiota of the vagina, *L. crispatus* was incubated at 2 and 24 h with KSFM medium, which supports the growth of human vaginal epithelial cells (12). Acting as a control, it was expected that under these conditions the bacteria would grow to a sufficient number of colonies. These plates were used as a maximum threshold to compare the serial dilutions done in the absence or presence of OTC vaginal products. The results from the series of incubations and dilutions concurred with the expectations for good support of bacterial growth demonstrating recovery of CFU input counts (Fig. 1 columns one). Acting as a bactericidal control, N-9 was incubated along with *L. crispatus* for both 2 and 24 h. As expected, N-9 had a negative effect on the bacteria. At 24 h N-9 completely killed the bacteria, while at 2 h, N-9 significantly (*p*<0.01) decreased the number of bacterial colonies (Fig. 1). The personal lubricant explored during this study decreased number of bacterial colonies by less than 1 log at 2 h incubation period (*p*<0.05) (Fig. 1A); however, at 24 h, it completely prevented bacterial growth and no colonies were visible from seeding of either diluted or undiluted bacterial suspensions (Fig. 1B). Vagisil significantly suppressed *Lactobacillus* growth even at a short 2 h incubation time (Fig. 1A) and at 24 h completely abolished bacterial colonies (Fig. 1B). The vaginal douche tested in this study did not show any significant effect on *L. crispatus* as compared to the KSFM control (Fig. 1A and B).

### Effect of products on bacterial-epithelial interactions

**Fig. 2:**
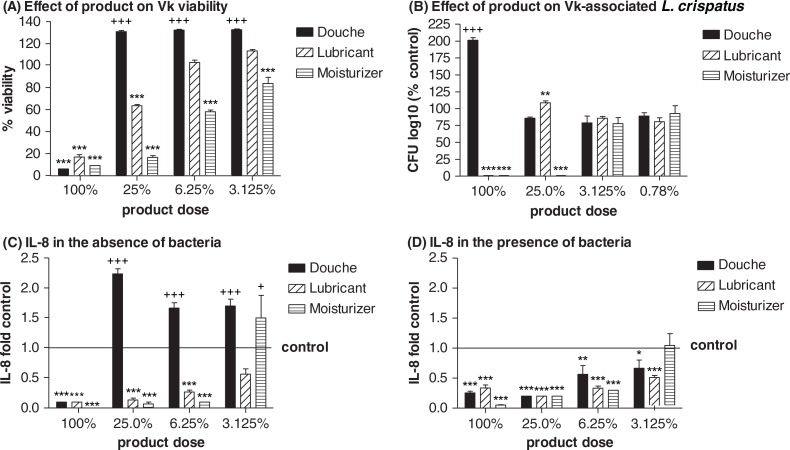
(A) Effect of product on vaginal keratinocyte (Vk) viability assessed by MTT assay, (B) *L. crispatus* colonization assessed by Vk-associated CFU, and Vk IL-8 production in (C) the absence or (D) the presence of *L. crispatus*. Data represent means and SEM of biological triplicates. * = *p*<0.05, ** = *p*<0.01, *** = *p*<0.001, values in product-treated cultures lower than medium control; + = *p*<0.05, + + + = *p*<0.001, values in product-treated cultures higher than medium control.

All products tested were toxic to the vaginal keratinocytes when applied undiluted (at 100% dose) (Fig. 2A). The douche was the least toxic and the moisturizer most toxic over a set of dilutions (Fig. 2A). While the undiluted douche stimulated bacterial growth (increasing recovery of epithelial cell-associated CFU to ~200%), the other products suppressed bacterial growth, with the moisturizer being suppressive up to a 4-fold dilution (at 25% dose) (Fig. 2B). The products had differential effects on the production of IL-8, which is an important proinflammatory innate immunity mediator produced by the vaginal epithelium to attract leukocytes to the mucosal surface in response to infection (18, 19). In the absence of bacteria the douche triggered increased IL-8 production (Fig. 2C). *L. crispatus* counteracted this effect. In the presence of *L. crispatus*, all products suppressed IL-8 production (Fig. 2D).

## Discussion

The mucosal lining of the vagina provides essential help in maintaining a healthy vaginal environment. The results from this experimental study demonstrate that some vaginal products may be harmful to the *Lactobacillus* bacteria and therefore should be used with caution. Referring to Fig. 1, both the lubricant and N-9 showed to have a significant effect on the growth of *L. crispatus*. At 24 h (Fig. 1), both the lubricant and N-9 completely inhibited bacteria growth in our experimental system.

The results from this experimental study confirm the hypothesis that some feminine hygiene products may alter the vaginal immune barrier by having a negative effect on the epithelial cell integrity (measured by MTT), survival of beneficial *Lactobacillus* species in the vaginal microenvironment (measured by epithelial-cell-associated CFU), and by changing the ability of the vaginal epithelial cells to produce protective or inflammatory immune mediators, for example, IL-8. The results confirm clinical findings of the harmful effects of certain vaginal hygiene practices on susceptibility to infections. The moisturizer and the lubricant were especially cytotoxic and negatively affecting *L. crispatus* survival. The douche, on the other hand, could promote a proinflammatory environment in the absence of *Lactobacillus* or when the microbiome is disturbed. More clinical studies are needed to test the findings and conclusions from these experiments, which if confirmed would argue for the benefit of using probiotics for vaginal health.

Our results provide experimental warning that women who have used nonoxynol-9, Vagisil, or lubricant may have a weakened vaginal barrier due to destroyed *L. crispatus* and perhaps other normal microflora species not assessed in our study, thus becoming at risk for the development of the syndrome of disturbed vaginal microbiome (20). Women using such products on a regular basis should take measures such as regular use of condoms to prevent sexually transmitted infections and should be evaluated for BV, which can sometime be asymptomatic and thus remain unnoticed by the vaginal product user. Although the particular douche kit tested did not show to have a negative effect on *L. crispatus* growth in our experimental system, it must be noted that some douching products have been associated with B streptococci and different *Candida* species (21). Douching has also been associated with a non-regression of low-grade squamous intraepithelial lesions (21). The use of vaginal spermicides and vaginal medications has been known to onset an unstable microflora and BV (22). It has to also be noted that the effects of vaginal products on bacterial growth can be selective and can vary among commercial brands, as suggested by another *in-vitro* study, which demonstrated differences among seven commercial douches tested against *Lactobacillus* isolates as well as BV associated bacteria (23). All three products tested were estimated to be fairly acidic (Table 1), although the pH varied within values typical for normal vaginal environment, except for the douche, which was more acidic.

Using the data that was amassed from this project, more research can hopefully go into vaginal health and development of probiotics for restoring and maintenance of a healthy vaginal microflora. Conventional health care wisdom has warned consumers against the use of douches a due to their stripping the vagina of its protective lining and altering its pH. Though the brand we tested did not show to inhibit the growth *in-vitro* of *L. crispatus*, frequent douching with this or other brands may throw the vagina's internal protective system off balance and promote non-sexually and sexually transmitted infections. Regardless of this, millions of females still continue to use it for personal and cultural reasoning. Hopefully this research may blossom into a new field in the biopharmaceutical market. Just as vaginal suppositories exist containing estrogen replacement therapies and anti-fungal agents to combat non-sexually transmitted infections such as candida (yeast) and BV, we may envision vaginal probiotic replacement therapies in the future in order to promote vaginal health and curb the frequency of reoccurring yeast infections and as a supplement available to women who continue to use these products. Preventative therapies are the path of modern medicine. Finding treatments for tomorrows’ disease may be the cloud with a silver lining for patients who are afflicted, but preventing them is golden.
